# Supplementary material for: The Gut Bacteria Dysbiosis Contributes to Chronic Graft-Versus-Host Disease Associated With a Treg/Th1 Ratio Imbalance
Source: Front Microbiol. 2022 Sep 8;13:813576. doi: 10.3389/fmicb.2022.813576 (PMC9493085; doi:10.3389/fmicb.2022.813576)
Supplement: Supplementary file 1 [file Data_Sheet_1.zip › P101SC18090073-01-B1-3-4_result/04.BetaDiversity/NMDS/NMDS.pdf]

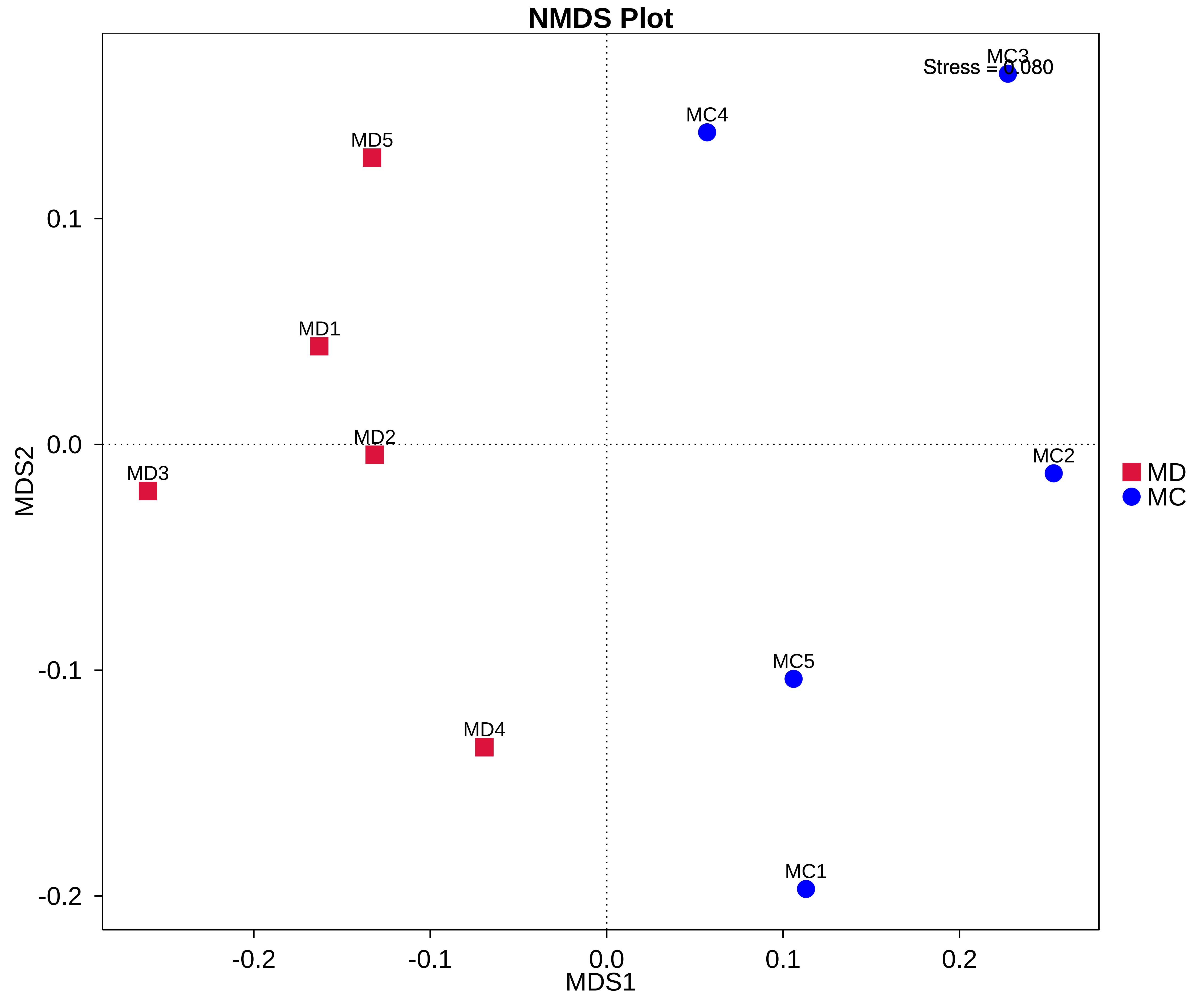
NMDS Plot
Stress = 0.080
MC3
MC4
MD5
MD1
MD2
MD3
MC2
MC5
MD4
MC1
MD
MC
0.1
0.0
-0.1
-0.2
-0.2
-0.1
0.0
0.1
0.2
MDS1
MDS2
